# Stable C and N isotope analysis of hair suggest undernourishment as a factor in the death of a mummified girl from late 19th century San Francisco, CA

**DOI:** 10.1371/journal.pone.0184921

**Published:** 2017-09-18

**Authors:** Jelmer W. Eerkens, Bryna Hull, Jena Goodman, Angela Evoy, Joshua D. Kapp, Sidra Hussain, Richard E. Green

**Affiliations:** 1 Department of Anthropology, University of California Davis, Davis, California, United States of America; 2 Department of Ecology and Evolutionary Biology, University of California Santa Cruz, Santa Cruz, California, United States of America; 3 Department of Biomolecular Engineering, University of California Santa Cruz, Santa Cruz, California, United States of America; University of Otago, NEW ZEALAND

## Abstract

The chance discovery of a 1.5–3.5 years old mummified girl presents a unique opportunity to further our understanding of health and disease among children in 19^th^ Century San Francisco. This study focuses on carbon and nitrogen stable isotope signatures in serial samples of hair that cover the last 14 months of her life. Results suggest an initial omnivorous diet with little input from marine resources or C4 plants. Around six months before death δ^15^N starts a steady increase, with a noticeable acceleration just two months before she died. The magnitude of δ^15^N change, +1.5‰ in total, is consistent with severe undernourishment or starvation. Cemetery records from this time period in San Francisco indicate high rates of infant and child mortality, mainly due to bacterial-borne infectious diseases, about two orders of magnitude higher than today. Taken together, we hypothesize that the girl died after a prolonged battle with such an illness. Results highlight the tremendous impacts that modern sanitation and medicine have had since the 1800s on human health and lifespan in the United States.

## Introduction

In May of 2016, a sealed metallic casket containing a mummified girl was found during construction activities in the Richmond District of San Francisco, California. The casket style indicates a burial date from the 1860s to late 1880s. Although many modern residents are unaware, this location in San Francisco was the site of a cluster of four cemeteries that operated mainly from the mid-1800s to early 1900s [1–3]. Detailed mapping shows that this particular girl was buried in the Independent Order of Odd Fellows Cemetery (IOOFC). Following growing complaints from nearby residents, and a desire to redevelop the area for housing to serve a growing city, IOOFC stopped accepting new interments in 1902, placing a maximum possible date for the casket.

In the early 1930s IOOFC was officially closed, and bodies systematically exhumed and transported south to the city of Colma, where the majority of individuals were reburied and remain today [4]. As exemplified by the casket, exhumation work was incomplete. Indeed, human remains and cemetery-associated artifacts (e.g., headstones, crosses) have occasionally been found in gardens, under streets, and in other areas of the former cemetery over the last 50 years [5–6]. Historic photos of the IOOFC exhumation show a systematic search and excavation strategy, but given the lack of detailed records of burial plots, it is not surprising that many individuals were missed.

While skeletal elements are occasionally found, the discovery of a preserved and complete mummified body is extremely unusual. The casket seal limited the availability of oxygen while the metal inhibited microbial activity. Combined with the cool climate of San Francisco, soft tissues were well preserved, though decomposition greatly accelerated after the casket was opened. Analysis of hair provides a unique opportunity to apply modern archaeometric and forensic techniques to understand more about diet and living conditions in San Francisco in the late 1800s. This paper describes the results of high resolution stable carbon and nitrogen isotope analyses on segments of hair, with the goal of providing insight into the girl’s diet and cause of death. In particular, we aim to examine whether she died of sudden causes, for example, due to an accident or acute infectious disease, or a more drawn-out chronic illness.

## “Miranda Eve”

The mummified girl was dubbed “Miranda Eve” by the home owner. This name was also used in popular media coverage reporting her discovery. Until genetic analyses (currently underway) confirm the true identity of the girl, we use that moniker to refer to her as well. No formal forensic or archaeological studies were conducted prior to reburial on June 4, 2016. However, the senior author was able to obtain two locks of hair, approximately 200 strands each, prior to reburial.

**Fig 1:**
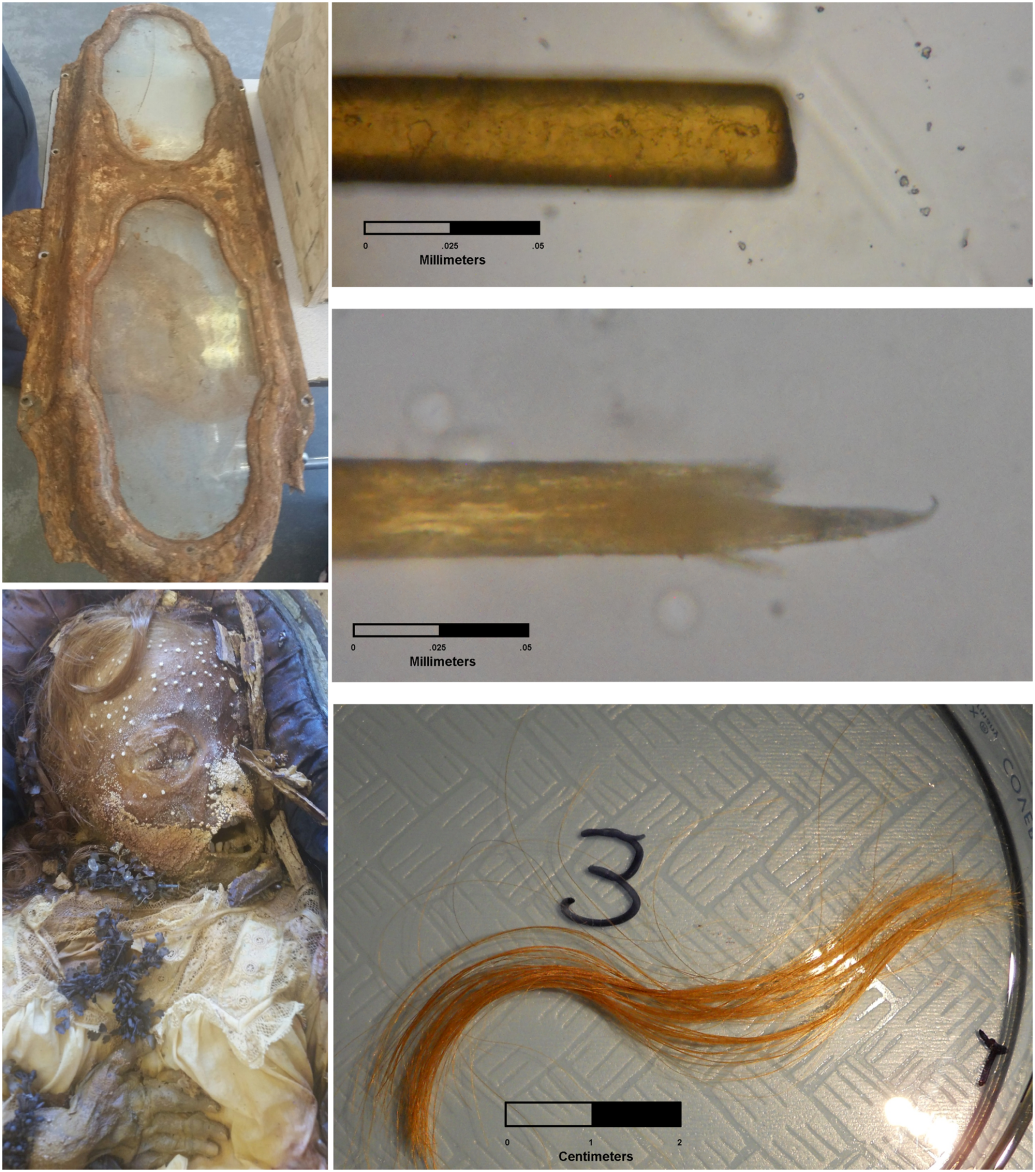
Metallic casket (upper left), Miranda Eve with floral cross (lower left), and photos of hair showing cut and root ends under magnification (right). Left-side photos courtesy of Elissa Davey.

The color and texture of the hair indicates that Miranda Eve was likely of European ancestry [7]. She was buried in a long white funeral dress and covered with many flowers, including one floral arrangement in the form of a cross (Fig 1). The metallic casket was an expensive brand. Together, this evidence suggests she was from an upper-class Christian family, and by 19^th^ century ethnic categories would have been classified as “white.” This is consistent with surviving IOOFC records, which indicate that the majority of internments were of European ancestry.

Several measures were used to estimate an age at death. First, fully erupted deciduous front and lateral incisors and canines indicate an age at death of at least 1.5 years, but earlier than 5–6 years when permanent teeth erupt [8]. Second, measurements on Miranda Eve indicate a stature of approximately 90 cm. Using 2006 World Health Organization charts, such a stature indicates an age of 1.8–3.5 years (95% confidence interval for girls). We used WHO information because that data includes children from a range of countries and socio-economic settings where children may be nutritionally challenged. In any case, given results presented below regarding nutritional deficiency, we acknowledge that Miranda Eve’s growth may have been stunted during the final months of her life, thus, the age estimate based on stature may slightly underestimate true age. Third, the average diameter of 10 of Miranda Eve’s hairs near the root is 0.027 mm. Hair diameter is known to increase with age, and this diameter suggests an age around 3 years, but perhaps as young as 2 or as old as 5 years given variation in hair diameter for girls of European ancestry [9–10] (note that age estimates are only given in 1-year increments in these studies). Taken together, we estimate her age at death between 1.5 and 3.5 years.

Based on the presence of a funeral dress and long hair, sex was initially assumed to be female. Indeed, the gender-specific name “Miranda Eve” assigned by the home owner and used in the press connotes a girl. However, such an assignment is based on modern notions of sex and visual expression of gender. Historical texts and photos show it was not uncommon for upper-class young boys to wear dresses and grow their hair long. As discussed below, we tested the sex of Miranda Eve based on genetic markers in the hair, and confirmed her sex as female. Therefore, we use female pronouns (she, her) when referencing Miranda Eve throughout.

## 19^th^ century mortuary context

To provide context to Miranda Eve, we examined cemetery records from late 19^th^ century San Francisco. We were interested in documenting common causes of death for children under the age of 4 years. Unfortunately, many official city records were burned in the 1906 fires following the famous earthquake. Likewise, while some IOOFC records are available, they lack sufficient detail and often do not list a cause of death.

By contrast, records from the neighboring Calvary and Holy Cross Cemetery (CHCC) are more systematic and complete. CHCC drew on a similar segment of San Francisco society, namely middle and upper class white citizenry, and we believe it provides a similar demographic to IOOFC. Hand-written records are available online for CHCC [11] and we encoded information for all internments from 1861 (n = 436), 1865 (n = 959), 1870 (n = 1509), and 1876 (n = 2304) to provide a mortuary context for Miranda Eve.

**Fig 2:**
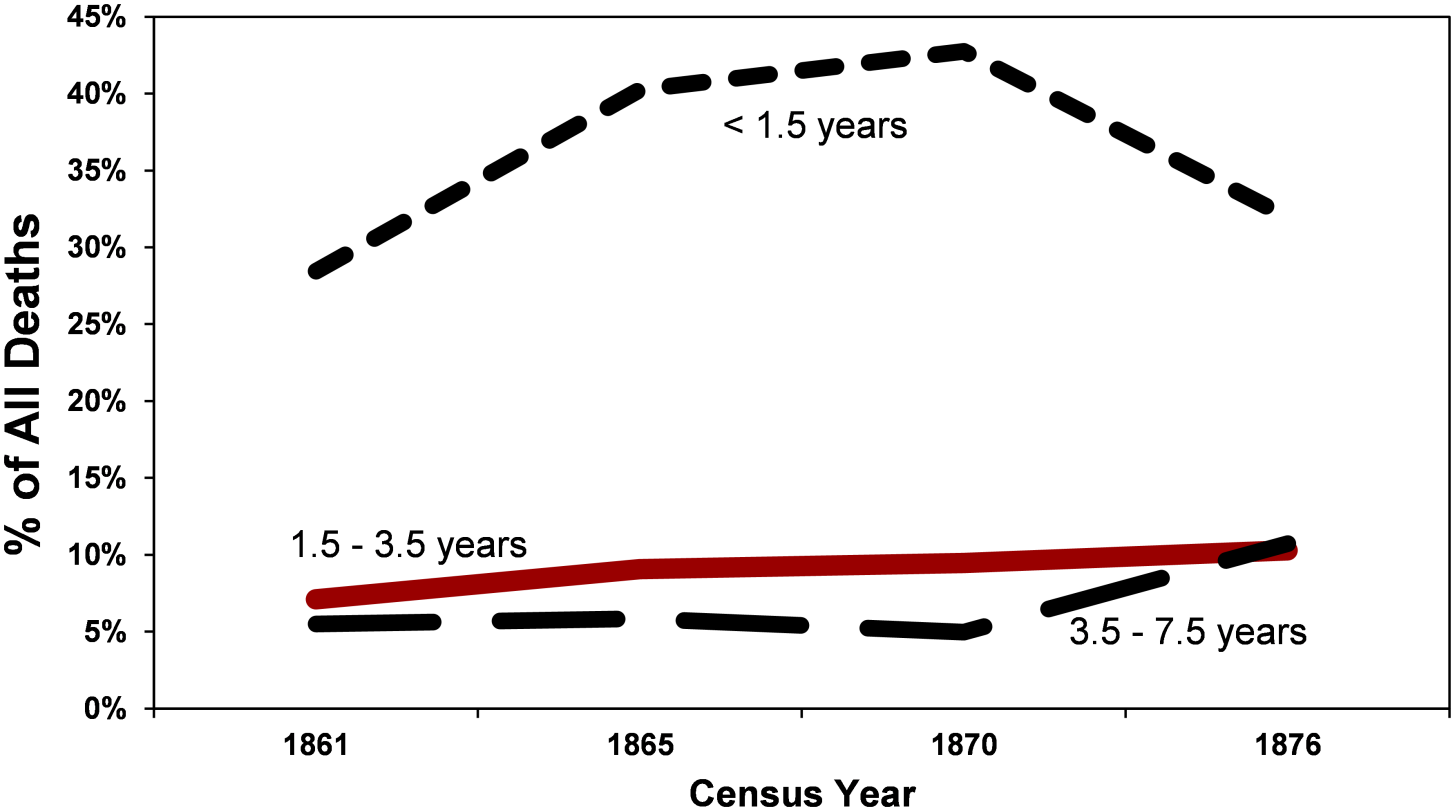
Percent of all deaths 1861–1876: Infants (<1.5 years), young children (1.5–3.5 years), and older children (3.5–7.5 years).

Within the records we examined, children account for a large fraction of all internments. The percentage of children under the age of 3.5 years varies between 36% (1861) and 52% (1870) of all internments, averaging 45% (Fig 2). Of these, the majority (80%) are under age 1.5 years. This is consistent with high rates of infant mortality, a phenomenon recorded across North America in the late 1800s [12–14]. Children between the ages of 1.5 and 3.5 years, the cohort to which Miranda Eve belongs, make up approximately 10% of all internments. By comparison, infants account for less 0.4% of all deaths in San Francisco County from 2010–2015, and children between the ages of 1 and 4 years less than 0.03% [15]. In this respect, while a child’s death today is rare (and tragic), such events were commonplace in the late 1800s, and the finding of a 1.5–3.5 year old child in an historic cemetery is certainly not unusual.

**Fig 3:**
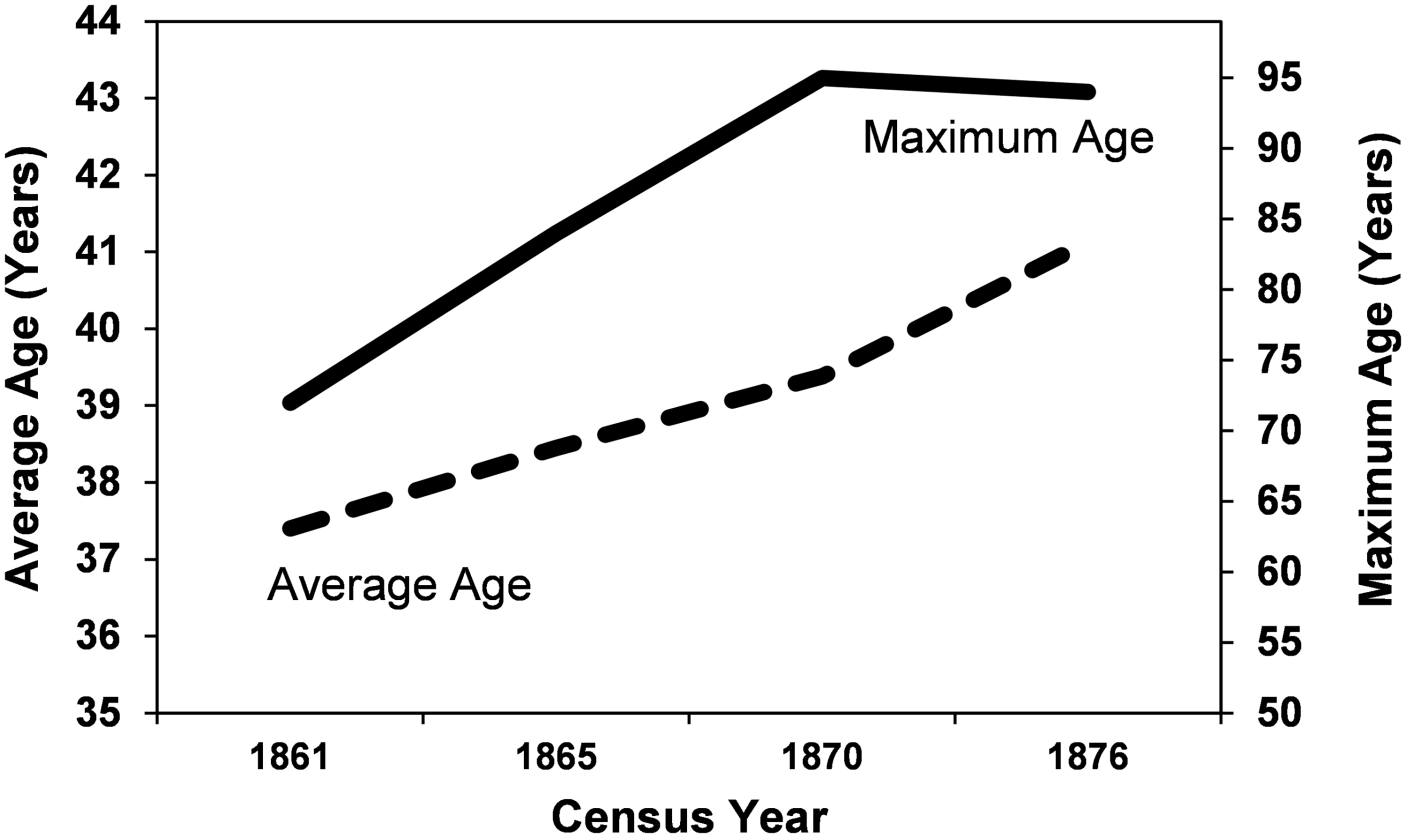
Maximum age (top solid line) and average age of individuals > 18 years (lower dotted line), 1861–1876.

While child mortality rates stayed roughly the same over time, there is some indication of increasing health in the adult population as measured by longevity (Fig 3). The average age at death for those over age 18 years, rose steadily from 37.4 to 41.1 years from 1861 to 1876, and the maximum age increased from 72 to 94 years over the same window. These changes may reflect increasing resistance to diseases and adaptation to urban life, or perhaps, better health care for ailments that afflicted adults (but, apparently, not children).

Within the CHCC records, we identified 498 children between the ages of 1.5 and 3.5 years for the four years under consideration. Of these, 450 have a legible “cause of death” listed (see Table 1). Medical understanding of health and disease was still in its infancy in the 19^th^ century. Many causes of death are what we would recognize today as symptoms (e.g., convulsions, congestion, debility, paralysis) of another underlying disease. Indeed, it is likely that some of the “causes” listed separately in Table 1 are really symptoms of another “cause” in the table. For example, diphtheria is an infection of the *Corynebacterium diphtheria* bacteria, but can cause the symptom “croup” (a type of barking cough associated with congestion of the upper respiratory tract), and croup was sometimes mistaken for whooping cough (pertussis), an infection by the *Bordetella pertussis* bacteria. Both diphtheria and whooping cough can ultimately lead to “convulsions” and death. We acknowledge that is difficult to separate (or combine) symptoms and diseases based on historical records alone and relate them to modern understandings of disease. In Table 1, we have combined recorded “causes of death” that today are recognized as symptoms, but are suggestive of a common underlying disease.

**Table 1:** Leading causes of death of children aged 1.5–3.5 years, 1861–1876.

Cause of Death, Age 1.5–3.5 years	Frequency 1861–1876	Length of Time
Diphtheria	15.6%	Acute
Croup	12.2%	Acute-Protracted
Scarlet Fever/Scarlatina	7.3%	Acute
Pneumonia/Lung Congestion	6.7%	Acute-Protracted
Meningitis	6.2%	Acute-Protracted
Brain: Congestion/Fever/Hydrocephaly	6.2%	Acute-Protracted
Whooping Cough	5.1%	Acute
Convulsions	5.1%	Acute
Accident	3.6%	Instantaneous
Small Pox / Variola	3.3%	Acute
Marasmus / Inanition	2.4%	Protracted

Diseases, and conditions likely caused by disease, dominate the list in Table 1. We also attempt to characterize whether death resulting from this cause would be instantaneous (within a day; as in a gunshot wound to the head), acute (1–7 days; as in a virulent illness), or protracted (over weeks to months; as in a chronic illness). In comparison to today, where accidents (e.g., drownings, car collisions) are the top cause of death among children, and deaths due to infectious disease are rare [15], accidents comprised a minority (less than 4%) and infectious diseases the vast majority from 1861–1876.

## Stable isotopes in hair

Human hair is composed mainly of keratin proteins that are synthesized within the body and secreted at follicle sites across the body. Typically, proteins from ingested foods are used directly to synthesize hair proteins. However, when dietary sources are deficient, the body will use alternative internal sources of protein, such as muscle, to synthesize hair. This catabolic process can have a fractionation effect on the ratio of different stable isotopes in the newly formed keratinous tissues [16–21].

The catabolic process has been best documented in the stable isotopes of nitrogen, in the ratio of ^15^N to ^14^N (expressed relative to an international standard as δ^15^N). δ^15^N is used in many stable isotope studies as a general marker of trophic level, with each trophic level (e.g., herbivores vs. carnivores vs. second-order carnivores) typically enriched by 2–4‰ [22]. Note that a breastfeeding child receiving a significant portion of their protein from breastmilk will also be enriched in δ^15^N relative to their mother, as it is higher in trophic level [23]. When individuals are under nutritional stress and their diet does not provide sufficient protein, they must catabolize amino acids from other bodily stores, such as muscle, to synthesize essential bodily tissues. This is similar to a trophic-level shift, and can cause δ^15^N in the newly synthesized tissue to increase up to 2‰ relative to values formed prior to stress. The effect has been observed, for example, in human hair of anorexic patients or individuals suffering serious nutritional deprivation [17, 20–21], as well as in non-human animals [18, 24–25], and is sometimes referred to as a “starvation signature.”

By contrast, carbon isotopes in keratin reflect the source of dietary carbon, in particular C3 (e.g., wheat, rice; depleted in ^13^C) vs C4 (e.g., maize, sorghum; enriched in ^13^C) plants, animals that are fed C3 or C4 plants, and/or marine foods (enriched in ^13^C) [22]. During nutritional shortfall, the ratio of ^13^C relative to ^12^C (expressed as δ^13^C) can also shift in hair, but because C is available in many more forms throughout the body (e.g., fat, muscle), the process is more complex [26–27]. In many cases δ^13^C decreases, but in some cases δ^13^C can increase. For example, in a study of hair from 16 individuals who died following an extended period of undernourishment, δ^13^C decreased in 11 of 16 and increased in the remaining 5, while δ^15^N increased in all 16 [21].

Because human hair grows sequentially over time, and is chemically stable once keratinized, it is possible to trace isotopically distinct sources of C and N used to synthesize hair. By sampling hair at different points along the shaft, we can trace changes in nutrient sources, including periods of undernourishment.

Hair samples from Miranda Eve were pulled from the scalp, preserving the root. Hair was examined under the microscope, and any visible foreign matter removed (e.g., foreign fibers). Samples were washed of residual surface materials by sonicating in deionized water. Eight bundles of approximately 10–15 strands each (80–120 total hair strands) were aligned at the root and cut into 5 mm serial sections using conventional scissors and freeze-dried. The last two sections were combined into a single 10mm long analyte to meet minimum weight requirements. Samples were then washed again in a 2:1 chloroform-methanol solution to remove lipids and other soluble materials, rinsed in dH_2_O three times, and placed in a 70°C oven to dry. Approximately 0.8mg of hair from the final 39 serial samples (last 20cm of hair growth) was submitted for δ^13^C and δ^15^N analysis at the Stable Isotope Facility at UC Davis using a PDZ Europa ANCA-GSL elemental analyzer interfaced to a PDZ Europa 20–20 isotope ratio mass spectrometer (Sercon Ltd., Cheshire, UK). Instrument precision is 0.1‰ and 0.2‰, respectively, for the two isotope measures based on repeated analyses of international standards.

Individual human hairs follow a cycle including an anagen phase where the hair is actively growing, and a telogen phase where the hair is still in place in the scalp but no longer growing. In healthy adults, about 90% of hair is in the anagen phase and 10% in the telogen phase [7, 28]. In children, the percentage of telogens is usually lower, between 1 and 10% [29]. On the other hand, the percentage of telogens is typically higher in stressed individuals [28, 30], due to premature termination of the anagen phase [31].

We were unable to establish the ratio of anagen to telogen phase hairs in the girl, nor were we able separate these prior to analysis. In spite of some noise from telogen hairs, by measuring δ^13^C and δ^15^N across ~80 hairs for each 5mm section we should capture a statistically representative measure of the source of Miranda Eve’s C and N at the time the majority of the hairs were growing. As well, while the contribution of telogen hairs may offset absolute values of δ^13^C and δ^15^N (the direction of the offset depending on earlier dietary history), provided they comprise only a minor portion of the total volume, changes in serial samples should preserve directional changes in diet.

Linear growth rates of hair in children are not well established. Though there is seasonal variation, controlled studies among adults of European ancestry suggests head hair grows at a rate of about 0.3–0.4 mm/day [32–33]. Children’s hair grows faster [33–34], though we are unaware of studies that calibrate the rate of growth specifically by age. Limited studies suggest a growth rate of around 0.4–0.5 mm/day. For this study we assume a rate of growth of 0.45 mm/day. Using this rate, the 20cm of hair analyzed represent the last 14 months of Miranda Eve’s life. Increasing the rate of growth would obviously decrease the amount of time represented in the strands of hair analyzed.

## Results

We extracted DNA from several strands of hair using standard ancient DNA protocols [35] in the UC Santa Cruz Paleogenomics lab clean room facilities. Shotgun sequencing libraries were prepared and sequenced on the Illumina MiSeq platform. Analysis of these sequence data from the mitochondrial genome (mtDNA) indicate that the DNA is largely devoid of contaminating DNA. The 95% confidence interval for the ratio of DNA sequences that match the Y chromosome to the number that match the X or Y chromosome [36], from 1,887,049 total sequences, is 0.0118–0.0134, indicating that the DNA is from a female.

**Fig 4:**
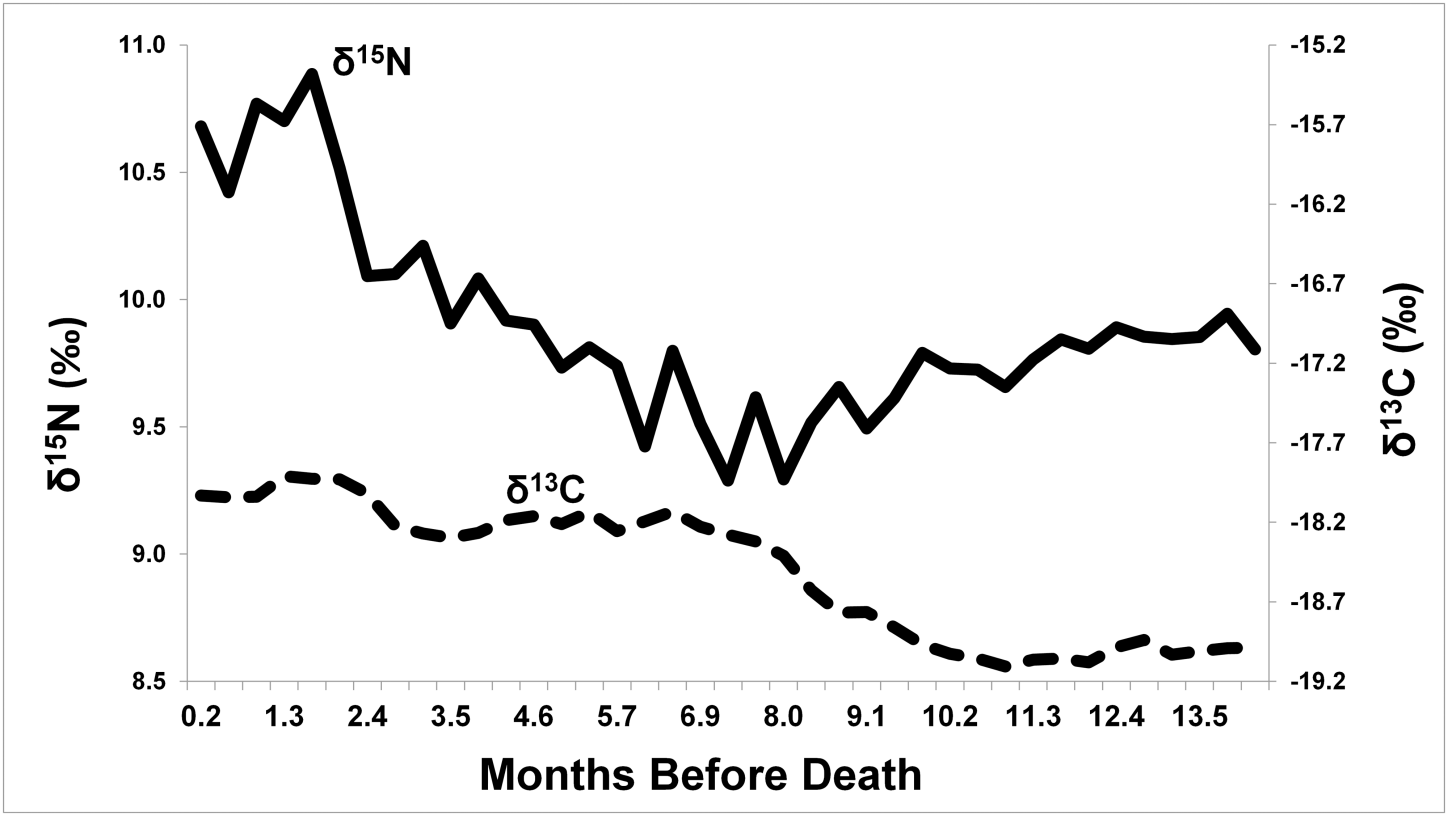
Serial δ^13^C (dotted line) and δ^15^N (solid line) from Miranda Eve hair segments.

Fig 4 shows δ^13^C and δ^15^N results for the 39 samples analyzed in this study (see raw data in S1 File). As shown, δ^13^C increases gradually in steps across the sequence, with a maximum difference of 1.2‰. Following relative stability from 14 to 10 months before her death, δ^13^C increases by about 1.0‰ between 10 and 7 months, is stable again between 7 and 3 months, and increases again by 0.4‰ between 2 and 3 months before her death. These shifts are well above instrument precision.

Changes in δ^15^N are more abrupt, showing a slow 0.7‰ decrease between 14 and 8 months before death, followed by a steady 0.8‰ increase between 8 and 2.4 months, and then a major jump of another 0.8‰ from 2.4 to 1.7 months before death, with values staying high thereafter. Maximum difference in δ^15^N is 1.6‰ across the serial samples, again, well above instrument precision.

## Discussion and conclusions

First, Miranda Eve is, as initially assumed, a girl. Second, starting values of δ^15^N (9.8‰) and δ^13^C (-19.0‰) are generally consistent with an omnivorous terrestrial diet, with little or no marine foods or C4 plants (e.g., maize; see results in [22, 37–38]). Third, changes across the serial hair samples are most consistent with undernourishment or starvation in the death of Miranda Eve. Following initial values through 8 months before death, δ^15^N slowly increases over a period of at least 5 months (from 7 months to 2.5 months before death). This is consistent with a phase of undernourishment where her bodily protein stores were slowly being consumed, resulting in steady enrichment of δ^15^N in hair keratin. At approximately 2.5 months, an acceleration in δ^15^N enrichment suggests a final phase that ended in death. This phase may have been marked behaviorally by a cessation of eating foods with significant quantities of protein. The slight increase in δ^13^C associated with this final phase may represent the introduction of small amounts of food, liquids (e.g., soup, broth), or medicines containing oils or carbohydrates that were incorporated in the production of hair, or may represent catabolization of internal sources of carbon. Similar isotopic patterns have been observed in sequential dental tissues of famine victims in Ireland [39]

Overall, isotopic data are inconsistent with an accident or acute disease (e.g., small pox) as a cause of death. Under such a scenario, we would expect little change in Miranda Eve’s diet in the months before death. Instead, the data are more consistent with a prolonged illness that used significant stores of her protein and energy. Chronic diarrhea may have limited nutrient uptake, or persistent illness may have reduced her appetite, ultimately resulting in starvation and death. Such a scenario is consistent with a bacterial infection, a common cause of death among children in late 19^th^ century San Francisco. Indeed, “inanition” and “marasmus” account for nearly 1 in 40 childhood deaths in the cemetery records we studied, and could have been caused by bacterial infections that interfered with eating and/or digestion of foods. Poor sanitation (especially water and sewage treatment), the lack of effective medical treatment (especially anti-bacterial medications), and a deficient understanding of disease transmission and the link between bacteria and human health, all contributed to high rates of bacterial-caused infectious disease across the United States [14]. Despite decades of discussion and planning, San Francisco did not invest in a city-wide sewage and water treatment system until the early 1900s [40]. Based on current evidence, we are unable to rule out other chronic conditions, such as leukemia, diabetes, or lead poisoning in the death of Miranda Eve. However, unsanitary conditions, and poor understanding regarding the transmission of pathogens from person to person, could have exposed Miranda Eve, like many children, to one or more infectious agents that contributed to her death.

In the final analysis, our examinations of cemetery records and stable isotope signatures in Miranda Eve’s hair underscore the dramatic effects that modern sanitation practices and medical science have had on human lives. Miranda Eve’s early death, while undoubtedly tragic for her family, was unfortunately a common experience for 19^th^ century families across the United States (and beyond). High infant and child mortality rates, caused especially by pathogenic bacteria, had tremendous social and biological effects on the lives of our ancestors. Indeed, we owe a tremendous debt today to these people who, in some manner, served as “guinea pigs” until patterns in disease (e.g., proximity to sewage, socio-economic class) and experiments with various chemicals (e.g., strychnine, bismuth), led governments and medical professionals to find solutions to reduce such suffering.

For the future, we are continuing work on extracting mitochondrial and nuclear DNA from Miranda Eve’s hair, with the hopes of characterizing her genetic ancestry in greater detail, and perhaps, matching her DNA to a modern living relative that might allow us to know her true identity. As well, we plan to examine her hair for isotopes of other elements (H and S) and potential traces of medicines that may have been administered during her final months of life.

## Postscript

Since the writing of this article, the research team has a positive identification on the girl. These results will be published separately, but girl has been identified through a DNA match to a living relative as Edith Howard Cook. Edith died on October 13, 1876, at age of two years, ten months and 15 days. This matches very well our original estimate of the age at death (1.5–3.5 years). More significantly, funeral home records list the cause of death as “marasmus.” As discussed above, marasmus is entirely consistent with the stable isotope pattern recorded in her hair, and further confirms her identity as Edith Howard Cook.

## Supporting information

S1 File δ^13^C, total C, δ^15^N, and total N measured in hair serial samples, along with hair segment distance from root (mm), and estimated median age of segment before death (months). (XLS)
